# Supplementary material for: Intraluminal infusion of Penta-Galloyl Glucose reduces abdominal aortic aneurysm development in the elastase rat model
Source: PLoS One. 2020 Aug 28;15(8):e0234409. doi: 10.1371/journal.pone.0234409 (PMC7454949; doi:10.1371/journal.pone.0234409)
Supplement: Attachment — Submitted filename: Response to Reviewers.docx [file pone.0234409.s001.docx]

Response to Reviewers

As response to the journal requirements, the points from 1 to 4 have been revised and corrected.

1. The manuscript has been corrected to meet the PLOS ONE’s style requirements.
2. In the methods section, additional information on the total number of animals used and how they were allocated to different experimental groups have been specified.
3. The financial statement has been removed from the Acknowledgments section and cover letter updated.
   I’d like to update the Funding Statement to the following: "Funded by Centre for Individualized Medicine in Arterial Diseases, Odense University Hospital". If further information regarding the funding is required, please let me know.
4. Regarding the data from the study. There are none legal or ethical restrictions regarding the data. Therefore, I have uploaded the data set necessary to replicate the findings. DOI: 10.17026/dans-zq2-ykhg.
5. While revising the submission, I have uploaded the figure files to PACE to help ensure that figures meet PLOS requirements. These new figures have been re-uploaded.

Reviewer’s comments to the author answered below.

### Reviewer #1 comments:

1. If possible, please provide evidence that penta-galloyl glucose does not precipitate with elastase.

Answer: When it comes to PGG precipitating with elastase, so far nothing has been described in the literature. Furthermore, in our experiments we flushed the vessels with saline prior to PGG administration se we would assume only minimal amount of elastase, if any, would still be present in the aortic lumen when PGG is administered.

To further elaborate it is important to know that several studies have detailed the chemical structure and functions of PGG (PMID: 20369791). When focusing on PGG’s effect on vascular tissue, its primary function is to utilize its proteins precipitation abilities done by hydrophobic forces and hydrogen bonding. Multiple methods of administration have been tested for PGG to evaluate its capabilities. An oral administration showed PGG to precipitate tannin-binding salivary proteins by forming hydrogen bonds and hydrophobic forces and inhibits its absorption in the alimentary canal. Has also been shown to be unstable in alkaline conditions, being chemically degraded in the intestines and having a low absorptive permeability.
Administrations by nanoparticles have shown that PGG is metabolized in the liver and easily forms insoluble precipitates (PMID: 30298373)

2. How the decision of PGG concentration was made?

Answer: The decision of using 0.06% (0.6mg/ml) as PGG concentration was based on both the perivasculary administration used in Isenburg et al’s (PMID:17372168) as well as the intravascular PGG concentration from Kloster et al. (PMID: 27144001).
The theory was that if PGG can penetrate the whole diameter of the aorta from the outside with that concentration, with minimal cytotoxic effects and an increasing trend in elastin preservation, then this concentration delivered intraluminally would also be able to penetrate layers of the aorta from the luminal side and out.

In regard of the PGG concentration Kloster et al. used, we compared the diameter of the aortic wall of their porcine model and the concentration of PGG used. This gave us a PGG concentration to diameter ratio, which we compared to the chosen concentration in Isenburg et al., which was almost the same.

3. What was the reason of 28 days study? The elastase induced AAA is known to develop in 1-2 weeks.

Answer: It is correct that the main development of AAA is happening in the first 1-2 weeks.
The 28 days was chosen both because Kloster et al. have showed a continued significant aneurysmal progression in pigs after the initial 1-2 weeks development. Isenburg et al. showed the AAA growth was increasing until day 28 and continued to growth until their endpoint at day 56. However, the growth rate seems to be dampened from day 28-56 and at the variation between rats seemed to increase. So, in order to detect an effect of PGG and limit the variation observed at day 56 in Isenburg et al. we chose to follow our rats for 28 days, while also wanting to bring further insight to the aneurysm expansion with our continued ultrasound controls.

4. The regulation of LOX was studied in the extracts derived from the whole aorta samples. The structure of the aorta, the ratio media/adventitia was dramatically changed in 28 days after AAA induction as shown by histology. Therefore, the regulation of LOX and CD45 mRNA is difficult to interpret. Could you provide immunohistological data about LOX Regulation in PGG Group?

Answer: Yes, we agree that it is very hard to interpret mRNA data from these heterogenous AAA wall samples. And support by histologically samples will clearly improve the papers quality and understanding. Due to the current covid19 situation we are unable to perform new experiments.

5. The innovation of this study should be more exactly formulated in the discussion.

Answer: Thank you, we have further specified the innovation of this study in the discussion with the following: “The DEB is a new and innovative method to administer PGG to a targeted location in an animal model. This approach was included in the hopes of providing the clinicians the tool to prevent continuous growth of small AAA, and type IA+B endoleaks after abdominal endovascular aneurysm repair (EVAR) by placing PGG in the aortic sealing zones by DEB.”

6. In Figure 5 (2) the graph is not understandable: Which one is PGG group and which is control?

Answer: Thank you, we have now updated the figure by enlarging the symbols and inserted the following in the figure legend: “The schematic development of dilatation in the infrarenal aorta was visualized in both the saline (triangle), control (square) and the PGG (circle) treated group, though no dilatations were seen in the saline treated group (E). Data are presented as mean ± SD.”

### Reviewer #2 comments:

This reviewer highlights several points:

1. The choice of PGG concentration is not explained, nor is the exposure time of 15 minutes.

Answer: The choice of PGG concentration is explained above in answer to question 2 from reviewer #1.
The chosen exposure time of 15 minutes was chosen both because Isenburg et al. used this exposure time and it seemed to be sufficient to be affective, but also because we wanted to shorten the time of the total ligation of the aorta for better survivability. In a study from 1998 by Yamaguchi et al. (PMID: 10383853) showed that a shorter infusion time reduces mortality rate.
We wanted to reduce the infusion time to below 1 hour. With the initial 30 minutes infusion of elastase/saline together with the time it takes to change catheters between infusions and the final ligation after the arterectomy we concluded that 15 minutes for the final PGG/saline infusion would be sufficient.

2. The Legend to Figure 4 is not clear - where are these sections from?

Answer: You are correct. We have improved the figure legend to the following for better understanding: “Histological slices of the abdominal aorta. The slices, A-C, were stained with Millers elastin stain, while the slices, D-F, were stained with Masson trichrome. Slice A and D were neighboring slices from same rat in the saline group, slice B/E were from the PGG group and C/F from the control group. TA: tunica adventitia. TM: tunica media. IT: intraluminal thrombus. Black arrows: marking of defragmentation. Dashed line: example of area with neovascularization and infiltrating leucocytes.”

3. Finally, I would remove porcine form the title - the source of elastase is irrelevant.

Answer: Porcine has been removed from the title. It now reads: “Intraluminal Infusion of Penta-Galloyl Glucose Reduces Abdominal Aortic Aneurysm Development in the Elastase Rat Model”
